# *Jasminum sambac*: A Potential Candidate for Drug Development to Cure Cardiovascular Ailments

**DOI:** 10.3390/molecules26185664

**Published:** 2021-09-18

**Authors:** Imran Ahmad Khan, Musaddique Hussain, Shaukat Hussain Munawar, Muhammad Omer Iqbal, Shafia Arshad, Ashira Manzoor, Mazhar Abbas Shah, Khizar Abbas, Waleed Shakeel, Shahzada Khurram Syed

**Affiliations:** 1 Department of Pharmacology, The Islamia University of Bahawalpur, Bahwalpur 63100, Pakistan; musaddique.hussain@iub.edu.pk (M.H.); oiqbal133@gmail.com (M.O.I.); 2 Department of Pharmacology and Toxicology, Cholistan University of Veterinary and Animal Sciences, Bahawalpur 63100, Pakistan; shaukathussainmunawar@cuvas.edu.pk; 3 Key Laboratories of Marine Drugs (Ministry of Education), Shandong Laboratory of Glycoscience and Glycoengineering, School of Pharmacy, Ocean University of China, Qingdao 266100, China; 4 Faculty of Medicine and Allied Health Sciences, The Islamia University of Bahawalpur, Bahawalpur 63100, Pakistan; shafia.arshad@iub.edu.pk; 5 Fatima Tu Zahara Department of Life Sciences, Muhammad Institute of Medical and Allied Sciences, Multan 60000, Pakistan; ashiramanzoor@gmail.com (A.M.); mazharshah558@gmail.com (M.A.S.); khizarabbasbhutta786@gmail.com (K.A.); 6 Department of Pharmacology, Bahauddin Zakariya University, Multan 60000, Pakistan; waleed.shakeel@yahoo.com; 7 Department of Basic Medical Sciences, School of Health Sciences, University of Management and Technology, Lahore 54770, Pakistan; shahzada.khurram@umt.edu.pk

**Keywords:** adrenaline, cardioprotective, CK-MB, AST, ALT, CRP

## Abstract

*Jasminum sambac* (L.) is a South Asian folkloric medicinal plant that has traditionally been used to treat cardiovascular problems. The current investigation was meticulously organized to explore the pharmacological foundation for the medicinal uses of *J. sambac* pertaining to cardiovascular ailments and to investigate the core mechanisms. Mechanistic investigation revealed that crude leaf extract of *J. sambac* produced ex-vivo vasorelaxant effects in endotheliumintact aorta ring preparation and hypotensive effect was recorded via pressure and force transducers coupled to the Power Lab Data Acquisition System. Moreover; *J. sambac* showed cardioprotective effects against adrenaline -induced left ventricular hypertrophy in rabbits observed hemodynamic. CK-MB, LDH, troponin, CRP, ALT, AST, ALP levels were shown to be lower in the myocardial infarction model, as were necrosis, oedema, and inflammatory cell recruitment in comparison to control. *J. sambac* has shown good antioxidant potential as well as prolonged the noradrenaline induced platelet adhesion. The vasorelaxant and cardioprotective effects in both in vivo and ex vivo experiments, which are enabled by activation of muscarinic receptor and/or releasing the nitric oxide and by reducing the adrenaline, induced oxidative stress, justifying its usage in cardiovascular disorders.

## 1. Introduction

Myocardial infarction is the leading cause of death in humans in many developed countries [1]. Because of the changing lifestyle patterns in many developing countries such as Pakistan, India, Bangladesh, and Afghanistan are rapidly catching up with this epidemic at an alarming rate [1]. Though synthetic medicines are very useful in the treatment of cardiovascular ailments their use is restricted due to their harmful effects [2,3]. Myocardial infarction is a serious ischemic situation in which extreme necrosis of myocardial tissue takes place. It happens because of the imbalance between blood supply and oxygen requirement of the cardiac muscles [4,5].

Adrenaline (ADR) is a catecholamine synthesized in the adrenal medulla. Adrenaline is a non-selective agonist of all adrenergic receptors consisting of alpha, beta and their subtypes primarily located on the cardiovascular system [6]. It causes myocardial infarction above the therapeutic dose (2 mg/kg body weight) [6]. This method is applied to induce myocardial infarction in the experimental animal to evaluate the cardioprotective effect of test drugs. It causes myocardial infarction because of lipid peroxidation that leads to the depletion of cellular antioxidants. It causes the overproduction of nitrosative derivatives oxidative stress occurs due to the overproduction of ROS [7]. It increases the Ca^++^ influx by opening the calcium channels of cardiomyocytes, increases oxidative stress by increasing the workload [8]. In a higher dose, it causes aortic and coronary vasoconstriction. It also promotes blood coagulation by increasing the level of clotting factor VIII, fibrinolysis as well as platelet count [9].

The importance of herbal medicines in the treatment of various ailments is invaluable globally. Herbal medicines are continuously providing important therapeutic agents not only in traditional but also in modern medicines and also because they are less toxic than synthetic medicines [3,5].

*Jasminum sambac* (Oleaceae), commonly called Jasmin, phytochemical investigation of *J. sambac* validated the existence of proteins, glycosides, coumarins, flavonoids, phenolics, resin, saponins, steroids, terpenes, ascorbic acid, essential oils and salicylic acid [10]. The dried leaf extract was found rich in quercitrin, quercitrin-3-dirhamnoglycoside, isoquercitrin, rutin, kaempherol-3-rhamnoglycosides, β-sitisterol, 2, 3-dihydro-benzofuran, α-amyrin,2, 6, 10-trimethyl, 1-nonadecene, bicycle-2,2,1-heptane-2,5-diol,14-ethylene-14-pentadecne, 1-nonadecene, alpha.-tocopherol-.beta.-d-mannoside, 1-heptacosanol. Jasmintides (jS1 and jS2), a unique plant based cysteine-rich peptides, were isolated from *J. sambac*, 1-Octadecyne; 1-Octadecyne; hexadecanoic acid, *n*-hexadecenoic acid, hexadecenoic acid, R-limonene, 9- octadecenoic acid, and squalene [11]. Pharmacological studies showed antimicrobial, analgesic, antipyretic, anticancer, antioxidant, antidiabetic, insecticidal, lipid-lowering, anti-obesity and gastroprotective effects [12]. Till now there is no recorded experimental foundation for its utilization as a cardioprotective agent. The present study was to see if hydroalcoholic leaf extract of *J. sambac* had any cardioprotective properties.

## 2. Results

### 2.1. Priliminary Phytochemical Screening

Preliminary phytochemical screening of *J. sambac* (Leaf) revealed the presence of saponins, coumarins, phenols, and flavonoids while alkaloids, tannins and anthraquinones were not detected during the phytochemical investigation (Table 1).

### 2.2. HPLC Analysis

**Figure 1:**
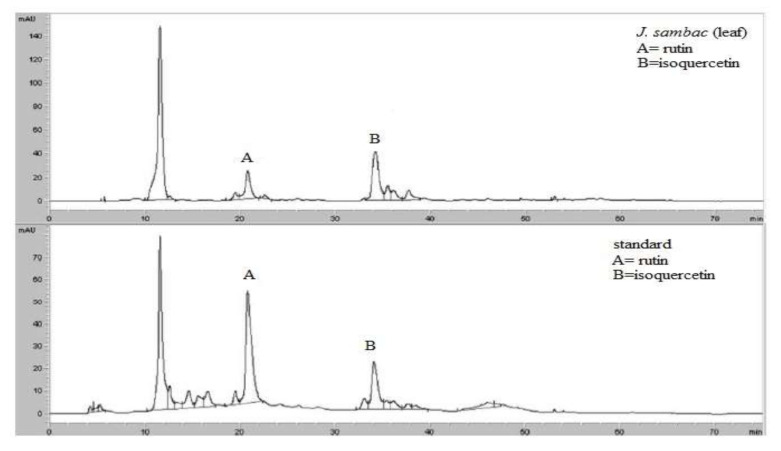
HPLC chromatogram of hydroalcoholic leaf extract of *J. samabac* showing rutin (A) and isoquercetin (B) similar to standard.

The analysis of High Power Liquid Chromatography (HPLC) revealed so many phytoconstituents in varing concentrations, in which the most prominent phytoconstituents are rutin and isoquercetin (Figure 1).

### 2.3. DPPH Assay

**Figure 2:**
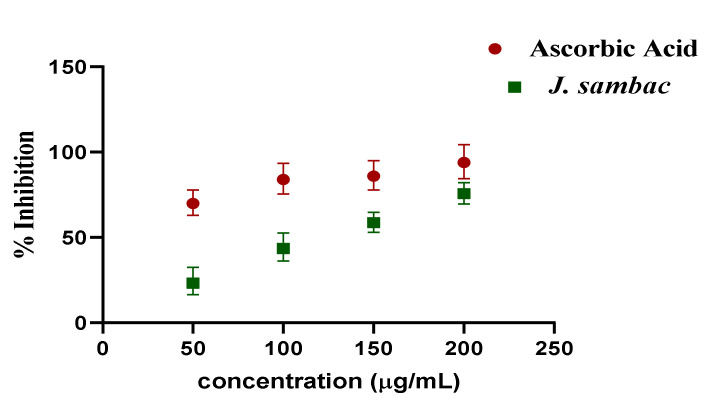
Antioxidant potential of hydroalcoholic leaf extract of *J. sambac* by DPPH assay.

In DPPH assay, the inhibitory concentration shown by the hydroalcoholic leaf extract of *J. sambac* was 120 µg/mL in comparison with ascorbic acid (Figure 2).

### 2.4. Acute Myocardial Infarction Study

**Figure 3:**
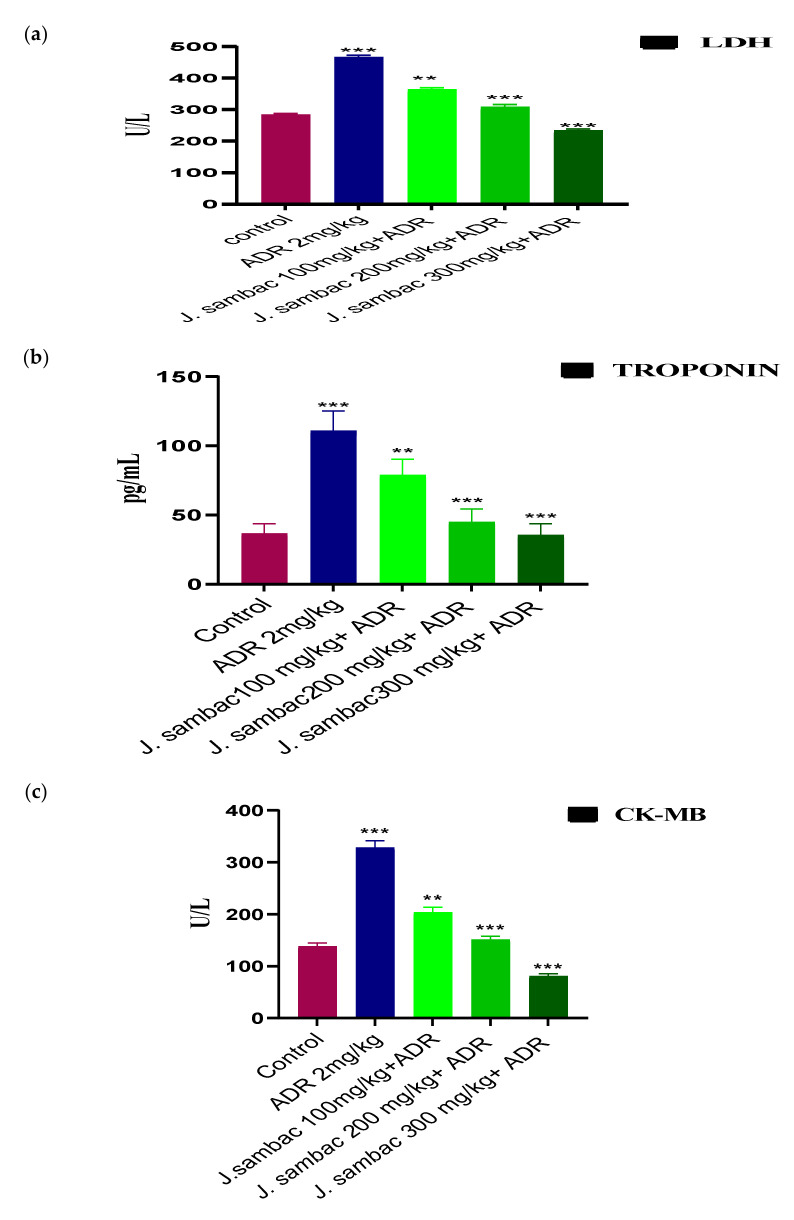
Cardioprotective effect of different doses of *J. sambac* extracts on the cardiac biomarkers, LDH (**a**), troponin (**b**) and CK-MB (**c**) against ADR-induced myocardial infarction in rabbits. One-way ANOVA was performed for the statistical analysis, comparisons among different groups was carried out by Dunnett’s multiple comparison test; ** *p* < 0.005 and *** *p* < 0.0001 (*n* = 5).

**Figure 4:**
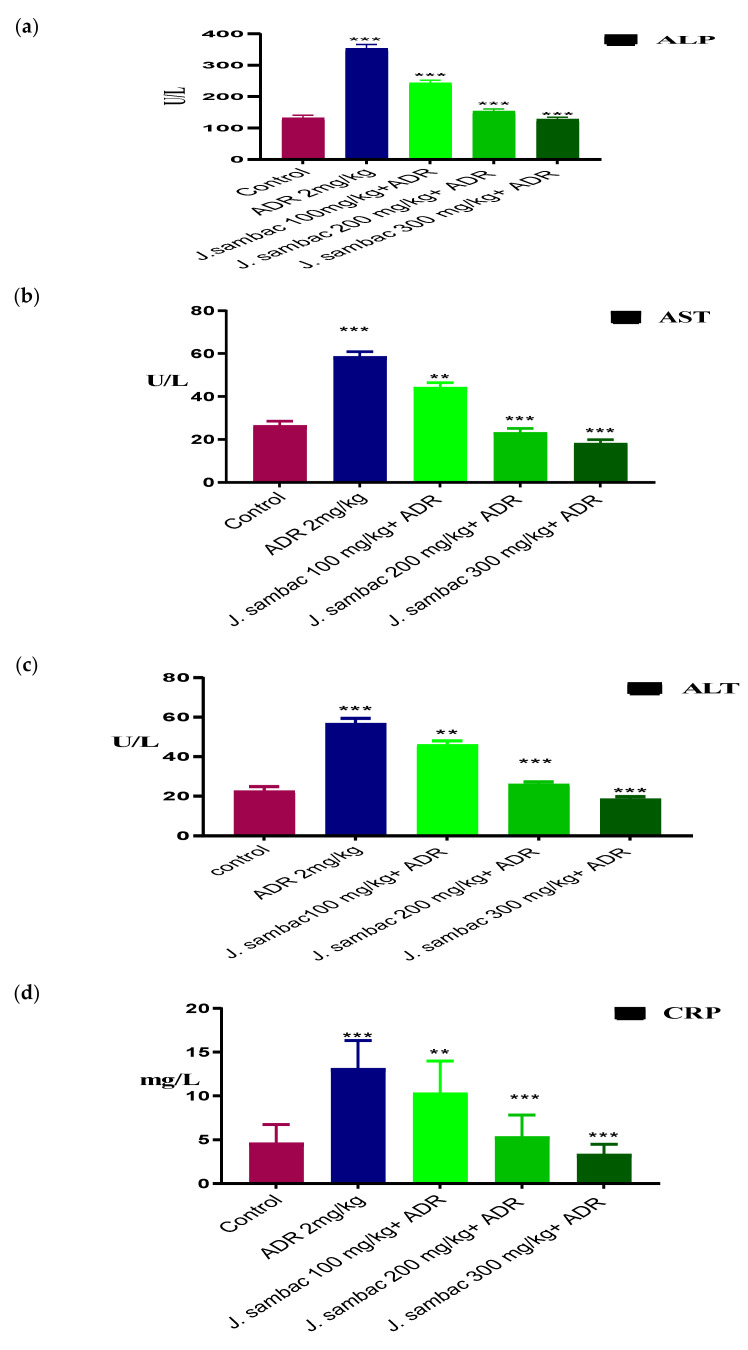
Cardioprotective effect of different doses of *J. sambac* extracts on the cardiac biomarkers, ALP (**a**), AST (**b**), ALT (**c**) and CRP (**d**) against ADR-induced myocardial infarction in rabbits. One-way ANOVA was performed for the statistical analysis, comparisons among different groups was carried out by Dunnett’s multiple comparison test; ** *p* < 0.005 and *** *p* < 0.0001 (*n* = 5).

ADR significantly increased the level of cardiac markers, CK-MB, LDH, troponin, CRP, ALT, AST, ALP (*p* < 0.05) concerning control. ADR group rabbits (group 2) had severe elevated cardiac markers, while the groups treated with the different doses of *J. sambac* showed resistance against the cardiac damage caused by the ADR in a dose-dependent fashion. In comparison, three groups receiving the leaf extract of *J. sambac* showed that the average amount of CK-MB, LDH, troponin, CRP, ALT, AST, ALP were reduced (*p* < 0.05) compared to those of the ADR group (Figure 3 and Figure 4).

#### Effect on Heart to Body Weight, and Weight of Heart to Tail Length Ratios

**Figure 5:**
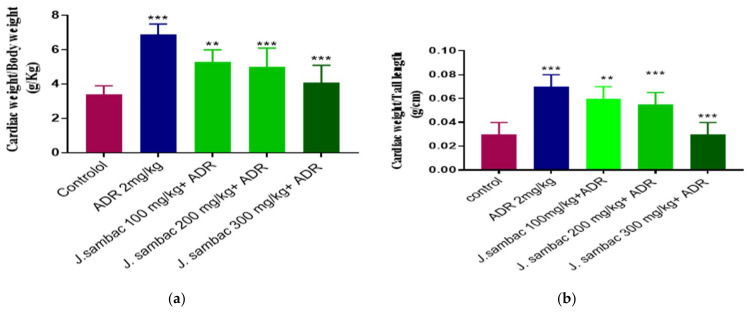
Cardioprotective effect ofdifferent doses of *J. sambac* on (**a**) cardiac weight to body weight ratio and (**b**) cardiac weight to tail length ratio in ADR-induced cardiac hypertrophied rabbits. One-way ANOVA was performed for the statistical analysis, comparisons among different groups was carried out by Dunnett’s multiple comparison test; ** *p* < 0.005 and *** *p* < 0.0001 (*n* = 5).

ADR significantly increased the ratios as compared to the control group. All groups treated with *J. sambac* were found to reduce the abovementioned ratios less than the ADR treated group (Figure 5).

### 2.5. Histopathology

**Figure 6:**
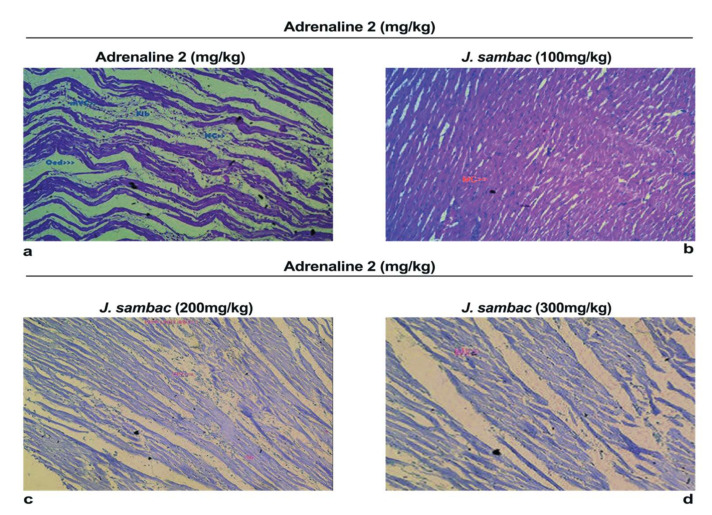
Photomicrographs of rabbit ventricle section from ADR-induced group (**a**) shows images from ventricle sections received *J. sambac* 100 mg/kg + ADR (**b**) shows images from ventricle sections received *J. sambac* 200 mg/kg (**c**) Shows ventricle sections received *J. sambac* 300 mg/kg + ADR (**d**). Fewer cardiomyocytes deterioration, fibrosis and less inflammatory cells were observed in dose dependent fashion in comparison with ADR intoxicated group.

Analysis of segments of heart tissues in the ADR-induced group showed a remarkable change in the cardiac cell structure. In ADR administrated group histological variations for example mononucleate cellular infiltration, interstitial oedema, disintegration and tear of muscular fibers, vacuolar disintegration, distention of capillaries, mottled staining, haemorrhage, and obstruction of the myocardium were observed. Severe necrotic lesions were noticed in all the rabbits while in *J. sambac* treated groups less myocardial deterioration was observed (Figure 6).

### 2.6. Isolated Aortic Tissue Preparation and Vasorelaxant Activity

**Figure 7:**
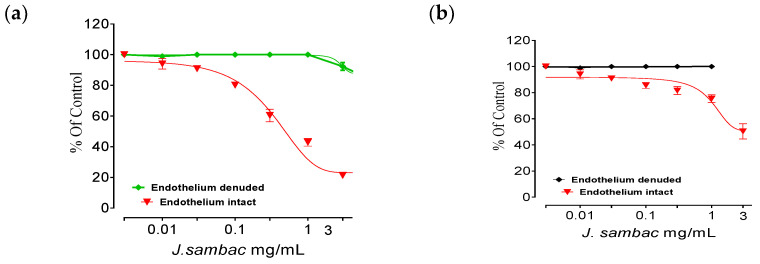

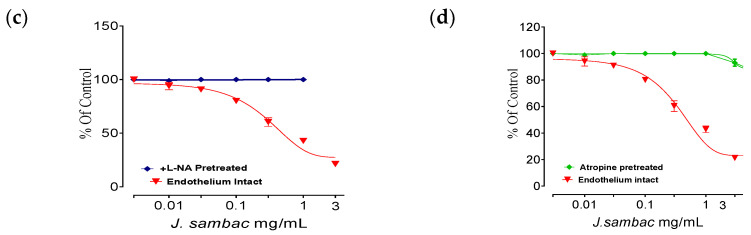
Effect of hydroalcoholic extract *J. sambac* against PE (1 μM) induced contraction on endothelium intact and denuded aorta (**a**), noradrenaline (10 µM) induced contraction (**b**), effect against PE (1 μM) induced contraction pretreated with L-NA (**c**) (1 × 10^−4^ M) and (**d**) atropine (1 × 10^−6^ M). All the values (*n* = 5) are depicted as mean ± SEM.

Phenylephrine (1 μM) caused a contraction in endothelium intact and denuded rabbit aorta. *J. samac* relaxed completely the endothelium intact aorta at a dose of 3 mg/mL, while at a similar dose, in endothelium-denuded aortic strip no vasodilation was recorded (Figure 7a). Noradrenaline (10 µM) caused vasoconstriction in endothelium intact and denuded rabbit aorta, but *J. sambac* produced partial vasodilation in endothelium intact aorta as compared to PE induced contraction at a similar dose while no vasodilatory effect was observed in endothelium-denuded aorta similar to PE induced contractions (Figure 7b). In L- nitro-arginine (1 × 10^−4^ M) and atropine (1 × 10^−6^ M) pretreated aortic strips, the vasodilator effect of *J. sambac* was disappeared at a similar dose (Figure 7c,d).

### 2.7. Effect on Adrenaline Induced Platelet Activation and Aggregation

**Figure 8:**
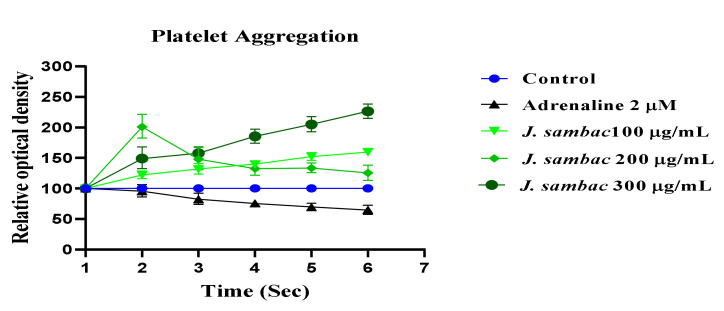
Effect of hydroalcoholic extract of *J. sambac* on the aggregation of platelet against ADR induced aggregation.

ADR (2 µM) addition to the suspension of washed human platelets produced a significant decline in the optical density at 600 nm, indicating the aggregation of platelets. The aggregatory effect was recorded at 37 °C. *J. sambac* (100, 200 and 300 µg/mL) dramatically inhibited the platelet aggregation (Figure 8) in a dose dependent manner.

### 2.8. Acute Oral Toxicity Dose Test

No mortality or morbidity was observed at any dose in acute oral toxicity dose test.

## 3. Discussion

As flowers and leaves generally have different phytochemical compositions, the majority of the published data is available on *J. sambac* flower and its essential oils biological activities [10]. ADR significantly increased the level of cardiac markers, CK-MB, LDH, troponin, CRP, ALT, AST, ALP (*p* < 0.001). In comparison, three groups receiving the leaf extract of *J. sambac* showed that the average amount of CK-MB, LDH, troponin, CRP, ALT, AST, ALP were reduced compared to those of the intoxicated group which advocates its cardioprotective potential (Figure 2). It was reported that the presence of high amounts of flavonoids in plants was responsible for their cardioprotective potentials [11,13] while, *J. sambac* leaf extract was found rich in flavonoids content (Table 1), [11]. Thus, it is logical to believe that the cardioprotective effect of *J. sambac* in rabbits might also depend on the presence of these flavonoids.

In the current study, the use of *J. sambac* hydroalcoholic leaf extract resulted in a significant reduction of CRP in groups receiving the extracts, which may be ascorbic acid- or flavonoid-based antioxidant effect of *J. sambac* (Figure 2) complimenting the previous antioxidant studies [5,14]. Saponins are believed to lower the level of lipid peroxidation products in a dose-dependent manner [15,16]. The results of this study add to the body of evidence supporting previously published studies on saponins or saponin-containing plant extracts’ cardioprotective effects in animal models [17,18]. Similarly *J. sambac* found richin saponins during phytochemcial evaluation (Table 1).

Early study [19] reported significant LPO inhibitory effect of hydroalcoholic leaf extract of *J. sambac* which further strengthens its claim as a cardioprotective agent as LPO is one of the major contributory factors in ADR induced MI [20].

Histopathology of heart tissues in the ADR-induced group showed remarkable damage in cardiac cell architecture for example; mononucleate cellular infiltration, interstitial oedema, disintegration and tear of muscular fibers, vacuolar disintegration, and distention of capillaries, mottled staining, haemorrhage, and obstruction of the myocardium were observed. Severe necrotic lesions were noticed in all the rabbits’ cardiac tissues but in *J. sambac* treated groups less myocardial deterioration was observed (Figure 6) which complements the results obtained from the biochemical investigation (Figure 3 and Figure 4). Less inflammatory cells were observed as compared with ADR intoxicated group which is evidence to its cardioprotective effect as reported in previous studies [21].

ADR increases the Ca^++^ influx by opening the calcium channels of cardiomyocytes, increases oxidative stress by increasing the workload [6]. A higher dose (2 mg/kg body weight), causes aortic and coronary vasoconstriction [6]. PE (1 μM) and noradrenaline (10 μM) causes vasoconstriction and increases the mean blood pressure primarily by stimulating the alpha-adrenergic receptor and/or opening of voltage-gated L-type calcium channels [22] which in turn increases oxidative stress (Figure 7). The vasodilator effect of *J. sambac* may depend on muscarinic receptor activation or release of cellular vasodilator nitric oxide because the vasodilatory effect was endothelium dependent and completely blocked when pretreated with atropine (1 × 10^−6^ M) and L- NA (1 × 10^−4^ M) like endothelium-denuded aorta (Figure 7). While partial vasodilator effect was observed against noradrenaline (10 µM)-induced aortic contractions, suggests the involvement of multiple pathways in its vasodilator effect [23]. Flavonoids-based vasodilator effect was reported in the plants earlier [24,25]. *J. sambac* found rich in flavonoid content (Table 1, Figure 1). Antithrombotic and anticoagulant drugs are well known source of cardioprotection in modern-day cardiology and ADR is a well-established procoagulant [6]. *J. sambac* significantly reduced the platelet aggregatory effect of ADR [Figure 8] which adds another colour to its cardioprotective potential.

## 4. Materials and Methods

### 4.1. Plant Materials

*J. sambac* had been collected fresh from Multan. It was authenticated by an expert botanist at the Institute of Pure and Applied Biology, BZ, University, Multan. The voucher specimen (R.R. Stewart, F.W. Pak. 549) has been placed for further reference.

### 4.2. Extract Preparation

The fresh plant was subjected to shade dry. All the foreign adulterants and vegetative waste were eliminated through manual picking before grinding the leaf part of the plant into a coarse powder with the assistance of a special herbal grinder. The crushed plant powder is stored in airtight jars waiting for extraction. The powder attained from only one batch was used in the experiment. *J. sambac* (250 g) leaf powder was soaking in a hydroalcoholic solvent (70:30 *v*/*v*) for 9 days in 2.5 L amber coloured air-tight jars. Then it was filtered and the filtrate was evaporated at 37 °C under reduced pressure on a rotatory evaporator to obtain a thick paste-like consistency [26]. At the end %age yield of the crude extract was calculated using this formula;

% age yield = Theoretical yield (gm)/Actual yield (gm) × 100(1)

### 4.3. Animals

Male rabbits weighing 1–1.5 kg had been obtained from the animal house of the Faculty of Pharmacy, The Islamia University of Bahawalpur. They had been nourished with standard commercially available food and tap water *ad libitum.* The temperature was maintained at 25 °C. The experiments were performed according to the rules of the National Institute of Health [27] and approved by the Ethical Committee of the Department of Pharmacology, The Islamia University of Bahawalpur, Pakistan (BOS & BOF/10/6/20).

### 4.4. Chemicals

Adrenaline, methanol, phenylephrine, and potassium chloride were purchased from Sigma-Aldrich, St. Louis, MO, USA. Verapamil was purchased from Searle Lahore, PB, Pakistan (PVT.) Ltd. (Lahore, Pakistan) AST, ALP, ALT, LDH, CRP Kits obtained from GM Chemicals and Diagnostics, Multan, PB, Pakistan. All other chemicals utilized the experiment were of highest purity and of reagent analytical grade.

### 4.5. Preliminary Phytochemical Evaluation

Phytochemical evaluation was done for the validation of various phytochemical classes (alkaloids, anthraquinones, glycosides, tannins, flavonoids and saponins) in the hydroalcoholic extract of *J. sambac* by using the standard protocol [28].

### 4.6. HPLC Analysis

Phytochemical compounds were separated by using the Agilent HPLC (Santa Clara, CA, USA) sequence 1100 system provided with autosampler, UV/vis detector, quaternary pump, 5 um, 250 mm × 4.6 mm i.d. and C18 reversed-phase column (Thermo Electron Corporation, Waltham, MA, USA) connect to HPChemStation software [29]. An (acetic acid–water, 2:98, *v*/*v*) and B (water–acetic acid, 2:98, *v*/*v*) were the solvents used to create the mobile phase (methanol). The following elution scenarios were used: 0–2 min, 5 percent B isocratic; 2–7 min, 5–25 percent B linear gradient; 7–11 min, 25 percent B isocratic; 11–19 min, 25–32 percent B linear gradient; 19–27 min, 32 percent B isocratic; 27–28 min, 32–40 percent B linear gradient; 28–38 min, 40 percent B isocratic. The solvents used to create the mobile phase, as well as the column’s cleaning and reconditioning procedures (38–50 min, linear gradient 40–100 percent B; 50–60 min, 100 percent B isocratic; 60–70 min, linear gradient 100–5 percent B; and 5 min, 5 percent B (isocratic). The prominent flavonoids in *J. sambac* leaves, rutin, and quercetin 3-d-glucoside were found had the following standardization curve:

A = 54,252 C − 100.12(2)

A = 87,077 C − 130.11(3)

where A is the area showed in mAu and C is the concentration showed in g/mL. The correlation coefficient (R2) was 0.9999 for both standardization curvatures. All the tests were performed in triplicate to have uniform results. HPLC chromatograms of the extract attained for two variations studied are shown in Figure 1.

### 4.7. Acute Oral Toxicity Dose Test

The acute oral toxicity of the extract was evaluated in 12 rabbits. They were divided into 3 groups and each group contained 4 rabbits and they were kept on fasting for 24 h and dosed in the following manner 500, 1000 and 2000 mg/kg body weight. After the dosing, the rabbits had been noticed for 14 days for lethargy, jerkiness, and death [30].

### 4.8. Determination of DPPH Assay

DPPH assay was conducted as described by using the previously described method [31]. In short, different concentrations of *J. sambac* extract (4 mL) was added to DPPH solution and prepared up to 5 mL with methanol, incubated for 40 min in dark. The spectrophotometer was used to measure the absorbance of the incubated solution at 517 nm. All of the tests were carried out in triplicate, and the percent inhibition was measured in ascorbic acid equivalents. The DPPH scavenging effect is calculated as:

1% = A (blank) − B (sample)/A (blank) × 100(4)

### 4.9. Acute Myocardial Infarction Study

The animals had been divided into 4 major groups each group contained 6 rabbits. Group-1 rabbit administrated ADR 2 mg/kg body weight, subcutaneously at 24 h of a gap for two successive days. Group-2 rabbits were pre-treated with 100 mg/kg of extract for the period of fourteen successive days and, adrenaline 2 mg/kg, was administrated on the 14th and 15th day at a gap of 24 h. The rabbits of group-3 were pre-treated with 200 mg/kg of extract for fourteen days consecutively and on the 14th and 15th-day adrenaline 2 mg/kg was administrated subcutaneously at a gap of 24 h. Group-4 rabbits were pre-treated with extract in an amount of 300 mg/kg for fourteen days successively and on the 14th and 15th-day, ADR 2 mg/kg was inoculated subcutaneously at an interval of 24 h while *J. sambac* doses were administered via oral gavages [32]. The three doses were selected based on previously reported data on the hepatoprotective potential of the *J. sambac* [33]. On 16th day, rabbits were anaesthetized, and the blood samples of rabbits taken from the marginal ear vein of the rabbits to assess the biochemical parameters, including CK-MB, LDH, troponin, CRP, ALT, AST, and ALP levels in serum, were measured by commercially available Kits.

#### Screening of Cardiac to Body Weight and Cardiac Weight to Tail Length Ratio

Cardiac weight and tail length were screened by measurement of cardiac weight to body weight help to key out cardiac weight index, the tail index was assessed by segmenting heart weight by tail length [6].

### 4.10. Histopathology

Rabbits had been killed under intense anaesthesia and the heart was isolated for histopathological examination, then the ventricular portion of the heart swiftly transferred to a 10% formalin solution. After that tissue had been submerged in the paraffin. A 5 μm thick segment had been cut and stained with a hematoxylin-eosin dye then mounted in the xylene [34]. Microscopic observation of the ventricular portion of the heart tissues from different groups was used to evaluate the adrenaline effect on the cardiac cells structure as well as its variation by the drug test inoculation under a compound microscope and micro-images were captured.

### 4.11. Isolated Aortic Tissue Preparation and Vasorelaxant Activity

Thorax cavity was opened, and carefully aorta was dissected out after slaughtering the rabbits. Fat and connective tissues were carefully detached from the aorta in a Petri dish containing Krebs’ solution. Aortic rings of 3–4 mm in length was cut and placed in tissue organ bath (10 mL) already contained the Krebs-Henseleit solution which is connected with thermocycler to maintain 37 °C and continuously bubbled with carbenogen gas (95% O_2_ and 5% CO_2_). One of the hooks was fixed to the bottom, and the other was connected to a force displacement transducer that connected to a Powerlab data acquisition system to record the isometric contractions. Kreb’ssolution was changed every 15 min to prevent the buildup of metabolites throughout the 40-min stabilization period at 1.0 g resting tension. After equilibration, the rings were preconstricted with 1 *×* 10^−6^ M phenylephrine (PE) until the steady contractile curve (5–8 min), and the vasodilator effect of *J. sambac* was recorded on cumulative dosing manner [35,36].

### 4.12. Effect on Adrenaline Induced Platelet Activation and Aggregation

The blood samples were collected from the marginal ear vein of the rabbit and centrifuged (3000 rpm for 15 min) to get platelet-rich plasma. Samples were supplemented with ADR (2 µM). Impedance aggregometry was used to assess platelet aggregation and flow cytometry was used to evaluate platelet activation before and after supplementations [37]. Firstly, the effect of three different added concentrations of adrenaline on platelet aggregation was assessed (*n* = 5). Next, platelet aggregation was measured after the addition of ADR in combination with *J. sambac* at 100, 200 and 300 µg/mL concentrations (*n* = 5).

## 5. Conclusions

The hydroalcoholic leaf extract of *J. sambac* was seen to produce vasorelaxant/hypotensive effect during ex-vivo study via stimulating the muscarinic receptors and/or releasing the local vasodilators NO. The cardioprotective effect of hydroalcoholic leaf extract *J. sambac* may be due to its variety of phytoconstituents. Pretreatment with *J. sambac* may refill cardiomyocytes with antioxidants, which are required for defense against the oxidative stress caused by adrenaline. However, the exact molecular mechanism of cardioprotection remains to be explored. Moreover, during phytochemical screening, tannins, flavonoids, and cardiac glycosides were discovered, which have a possible impact in cardiovascular disorders, particularly hypertension-induced ventricular hypertrophy and myocardial infarction. Conclusively, ex-vivo and in-vivo research revealed *J. sambac*’s therapeutic potential in cardiovascular diseases.

## Figures and Tables

**Table 1:** Phytochemical analysis of hydroalcoholic leaf extract of *J. sambac*.

Tests	Observation	Results
Alkaloid	no ppt	−
Saponins	1 cm froth	+
Tannins	No Light purple	−
Anthraquinones	No Pink Colour	−
Coumarins	Yellow fluorescence.	+
Phenols	Light purple	+
Flavonoid	Light yellow colour	++

+ = Present, ++ = highly present, − = absent.

## Data Availability

Not applicable.

## Sample Availability

Samples of the compounds are available from the authors.

## References

[1] Khan I.A. (2020). Pharmacotherapeutic modifications in cardiopulmonary patients during COVID-19 outbreak. J. Coll. Phy. Surg. Pak..

[2] Quiñones M., Miguel M., Aleixandre G. (2013). Beneficial effects of polyphenols on cardiovascular disease. Pharmacol. Res..

[3] Shaito A., Thuan D.T.B., Nguyen T.H.D., Hasan H., Halabi S., Abdelhady S., Nasrallah G.K., Pintus G. (2020). Herbal Medicine for Cardiovascular Diseases: Efficacy, Mechanisms, and Safety. Front. Pharmacol..

[4] Kumar S.V., Saritha G., Fareedullah G. (2010). Role of antioxidants and oxidative stress in cardiovascular diseases. Ann. Biol. Res..

[5] Tsoupras A., Lordan R., Zabetakis I. (2018). Inflammation, not Cholesterol, Is a Cause of Chronic Disease. Nutrients.

[6] Amin M.M., El-Gazayerly O.N., Gawad N.A., Halim S.M. (2016). Effect of formulation variables on design, in vitro evaluation of valsartan SNEDDS and estimation of its antioxidant effect in adrenaline-induced acute myocardial infarction in rats. Pharmaceut. Dev. Technol..

[7] Fathiazad F., Matlobi S., Khorrami N. (2012). Phytochemical screening and evaluation of cardioprotective activity of ethanolic extract of *Ocimumbasilicum* L. (basil) against isoproterenol induced myocardial infarction in rats. DARU J. Pharma. Sci..

[8] Saqib F., Arif Aslam M., Mujahid K., Marceanu L., Moga M., Ahmedah H.T., Chicea L. (2020). Studies to Elucidate the Mechanism of Cardio Protective and Hypotensive Activities of Anogeissus acuminata (Roxb. ex DC.) in Rodents. Molecules.

[9] Koffuor G.A., Amoateng P. (2012). Anti-oxidant and anticoagulant properties of *Phyllanthus fraternus* GL webster (Family: Euphorbiaceae). J. Pharmacol. Toxicol..

[10] Khare C.P. (2004). Encyclopedia of Indian Medicinal Plants, Rational Western Therapy. Ayurvedic and Other Traditional Usage, Botany.

[11] Al-Snafi A.E. (2018). Pharmacological and therapeutic effects of *Jasminum sambac*—A review. Indo Am. J. Pharma. Sci..

[12] Phanukit K.U., Chuleratana B., Kajsongkram T., Amonrat K.H. (2012). Chemical composition, toxicity and vasodilatation Effect of the flowers extract of *Jasminum sambac* (L.) Ait. “G. Duke of Tuscany”. Evid.-Based Complement. Altern. Med..

[13] Bucki R., Pastore J.J., Giraud F., Sulpice J.C., Janmey P.A. (2003). Flavonoid inhibition of platelet procoagulant activity and phosphoinositide synthesis. J. Thromb. Haemost..

[14] Kumari G., Wong K.U., Serr A., Joon S., Yoon S.H., Tam P. (2018). Molecular diversity and function of jasmintides from *Jasminum sambac*. BMC Plant. Biol..

[15] Dong Y., Duan L., Chen H.W., Liu Y.M., Zhang Y. (2019). Network pharmacology-based prediction and verification of the targets and mechanism for *Panax notoginseng* saponins against coronary heart disease. Evid.-Based Complement. Altern. Med..

[16] He Y., Hu Z., Li A., Zhu Z., Yang N., Ying Z. (2019). Recent advances in biotransformation of saponins. Molecules.

[17] Sun J., Yu X., Huangpu H., Yao F. (2019). Ginsenoside Rb3 protects cardiomyocytes against hypoxia/reoxygenation injury via activating the antioxidation signaling pathway of PERK/Nrf2/HMOX1. Biomed. Pharmacother..

[18] Mundugaru R., Udaykumar P., Senthilkumar S., Bhat S. (2018). Cardioprotective activity of fruit of *Garcinia pedunculata* on isoprenaline-induced myocardial infarction in rat. Bangladesh J. Pharmacol..

[19] Kalaiselvi M., Narmadha R., Ragavendran P., Arul R., Sophia D., Ravi K.G., Gomathi D., Uma C. (2011). In vivo simulated in vitro model of *Jasminum sambac* Linn. using mammalian liver slice technique. Asian Pac. J. Trop. Biomed..

[20] Ittagi S., Merugumolu K.M., Siddamsetty S.K. (2014). Cardioprotective effect of hydroalcoholic extract of *Tecoma stans* flowers against isoproterenol induced myocardial infarction in rats. Asian Pac. J. Trop. Biomed..

[21] Panchal S.K., Brown L. (2019). Cholesterol versus Inflammation as Cause of Chronic Diseases. Nutrients.

[22] Gilani A.H., Janbaz K.H., Zaman H., Lateef A., Suria A. (1994). Possible Presence of Calcium Channel Blocker(s) in *Rubia cordifolia*: An Indigenous Medicinal Plant. JPMA.

[23] Prince P.S., Rajakumar S., Dhanasekar K. (2011). Protective effects of vanillic acid on electrocardiogram, lipid peroxidation, antioxidants, proinflammatory markers and histopathology in isoproterenol induced cardiotoxic rats. Eur. J. Pharmacol..

[24] Wang X.L. (2015). Potential herb-drug interaction in the prevention of cardiovascular diseases during integrated traditional and western medicine treatment. Chin. J. Integr. Med..

[25] Satoh S., Nishida S. (2004). Electropharmacological actions of Ginkgo biloba extract on vascular smooth and heart muscles. Clin. Chim. Acta.

[26] Khan I.A., Lodhi A.H., Munawar S.H., Manzoor A., Manzoor Z., Raza M.A., Iqbal O. (2020). Assessment of ameliorative effect of Aab-e-Shifa polyherbal formulation in experimentally-induced wound in rabbits. Trop. J. Pharm. Res..

[27] National Institute of Health (1985). Guide for the Care and Use of Laboratory Animals.

[28] Trease G.E., Evans W.C. (2009). Trease and Evans’ Pharmacognosy.

[29] Teresa F.M., Lourdes C., Mantell C., Miguel R., Enrique M.O. (2012). Extraction of antioxidant compounds from different varieties of *Mangifera indica* leaves using green technologies. J. Sup. Fluids.

[30] Al-Afifi N.A., Alabsi A.M., Bakri M.M. (2018). Acute and sub-acute oral toxicity of Dracaena cinnabari resin methanol extract in rats. BMC Complement. Altern. Med..

[31] Sreejayan A., Rao M.N. (1996). Free radicals scavenging activity of curcuminoids. Drug Res..

[32] Salma A., El-Marasy S.A., Awdan E., Hassan H., Heba M.I. (2020). Cardioprotective effect of thymol against adrenaline-induced myocardial injury in rats. Heliyon.

[33] Veeresh B.D., Ramesh K., Bhatt K. (2017). Evaluation of Hepatoprotective activity of *Jasminum sambac* in rats. Int. J. Res. Pharmacol. Pharmacother..

[34] Khan I.A., Lodhi A.H., Munawar S.H., Manzoor A., Manzoor Z., Raza M.A. (2018). Formulation and evaluation of allicin and curcumin gel improves normal and diabetic ulcers in rabbits. Lat. Am. J. Pharm..

[35] Imtiaz S.M., Aleem A., Saqib F., Ormenisan A.N., Elena Neculau A., Anastasiu C.V. (2019). The Potential involvement of an ATP-Dependent Potassium Channel-Opening Mechanism in the Smooth Muscle Relaxant Properties of *Tamarix dioica* Roxb. Biomolecules.

[36] Isik B.S., Altay F., Capanoglu E. (2018). The uniaxial and coaxial encapsulations of sour cherry (*Prunus cerasus* L.) concentrate by electrospinning and their in vitro bioaccessibility. Food Chem..

[37] Singh S., Malm C.J., Ramström S., Hesse C., Jeppsson A. (2018). Adrenaline enhances in vitro platelet activation and aggregation in blood samples from ticagrelor-treated patients. Res. Pract. Thromb. Haemost..

